# Hospital Meetings

**Published:** 1896-03-28

**Authors:** 


### HOSPITAL MEETINGS.

### HOSPITAL AND HOME FOR INCURABLE CHILDREN. ANNUAL MEETING.

The twenty-first annual meeting of the Hospital and Home for Incurable Children was held at 2, Maida Vale on March 14th, the Rev. Canon Duckworth, D.D., vice-president, being in the chair. The minutes of the last annual meeting were read and approved.

The report of the committee was read, and its adoption, with the audited statement of accounts, was proposed from the chair, seconded, and carried unanimously. Satisfaction was expressed at the increased grant received from the Hospital Sunday Fund in 1895. The Chairman felt it a relief to turn from the wars and rumours of wars—the stirring events of the outside world—to the Home at 2, Maida Vale, where peace prevailed and sensational events were rare, although there were elements of tragedy in many of the young lives there. Of the work of the matron, Miss Coleman, Canon Duckworth spoke with enthusiasm. He considered that the thanks of all were due to her for the untiring care and wisdom she exhibited. It was impossible to overstate the value of her administration. She was in fact an institution in herself, and he could not possibly imagine what they should do without her. The resignation of Mr. Stanley Fisher, the hon. secretary, was a cause of great regret to the committee, who realised the difficulty of finding a successor to undertake the work which he had carried on so well for over three years, and now gave up owing to his inability to spare time from his own increasing work.

Mr. Hoblyn also spoke of the serious loss Mr. Stanley Fisher was to them, and hoped that he would be induced to accept a seat on the board.

A cordial vote of thanks to the hon. medical officer (Dr. F. E. Webb) was proposed by General Lowry, who spoke of the many ways in which Dr. Webb showed his unceasing interest in the institution; and also to the hon. chaplain for devoting much time to the patients. He included in the vote of thanks the matron and her admirable staff, and also those lady visitors who did so much to brighten the lives and add to the interests of the little patients.

At the conclusion of the meeting most of the visitors inspected the hospital, and were entertained at tea by the matron.

### THE NORTH LONDON HOSPITAL FOR CONSUMPTION AND DISEASES OF THE CHEST.

### Annual Court of Governors.

The annual court of the North London Hospital for Consumption was held in the board-room, Fitzroy Square, on March 18th, the chair being taken by Mr. F. D. Mocatta, Amongst those present were Mr. B. A. Lyon and Miss Lyon, Mr. D. F. Malcolm, General Young, the Rev. S. B. Burnaby, Mrs. Johnston, Mr. W. S. Gand, Mr. W. Titnas, Mr. J. S. Fletcher, Mr. Henry Stedall, Miss Flora Goldsmid, Mr. Alfred Essex, and others.

The adoption of the report was moved and carried. Mr. Mocatta considered the report was a satisfactory and clear composition, and remarked that the out-patient department was well attended, but not too crowded. He was also pleased to find that in a number of cases extension of time had been granted to in-patients, a beneficial arrangement specially appreciated in the winter months. He rather distrusted high figures in connection with hospitals, preferring that fewer persons should have efficient treatment rather than double as many of only partial relief. He acknowledged very cordially the obligations of the hospital to the hon. medical officers for their valuable and constant attendance. Thanks were also due to the ladies and gentlemen who did so much for the amusement of the patients in giving them concerts and entertainments. The resignation of their secretary (Mr. Hill) after eleven years' service had been received with considerable regret. His place had been filled by Mr. Bosworth, who had considerable experience in hospitals. Mr. Mocatta remarked that as usual they had cause to be grateful to Mr. Burdett, the grants this year from the Sunday and Saturday Hospital Funds being largely in excess of any former occasion. Mr. Burnaby regretted the absence of congregational collections, and advised the secretary to give this matter his attention, and endeavour to get ministers to grant them offertories, by which means a vast number of persons would be attracted to the merits of the charity. Mr. Lyon thought people might be trusted to give in the way they liked best, whether donations, subscriptions, or legacies, all being acceptable.

The auditors were reappointed. The election as vice-presidents of Mr. Noel E. Buxton and Captain Saunders was moved by Mr. Lyon, seconded by Mr. J. S. Fletcher, and carried. Mr. Lionel F. Hill, late secretary, and Mr. Henry Stedall were elected members of Committee of Management.

### LONDON TEMPERANCE HOSPITAL.

Mr. T. V. Strong presided at the annual meeting of the London Temperance Hospital held on Thursday, March 19th, in the absence of the Duke of Westminster, the president. The report adopted by the governors stated that the number of in-patients exceeded that for last year by 22, being 1,066. In the out-patients' department there had been 5,842 new cases, and a great increase had been observable in the casualty department, the number of cases being for 1895 8,826, as against 5,965 in 1894. A contemplated extension of the hospital was announced. At a public meeting held later on, Canon Fleming moved a resolution expressing a hope that the claims of the institution might receive a wide and practical response. He urged that support ought especially to be given to this hospital, because it had helped to prove that drunkenness was not a mere vice but a disease, and that "the use of alcohol was utterly needless under any circumstances to any person, and was absolutely injurious." The resolution was seconded by Mr. Whittaker, M.P., and carried. Afterwards speeches were made by Dr. Dawson Burns, Dr. Collins, Dr. Ridge, and Miss Orme (matron).

### POPLAR HOSPITAL FOR ACCIDENTS.—ANNUAL GENERAL MEETING.

The annual general meeting of governors was held at the Poplar Hospital on March 24th. The chair was taken by the Hon. Sydney Holland, who, in moving the adoption of the balance-sheet and report, drew attention to the very great progress made by the hospital. He regretted the small number of legacies received during the year, but realised that the hospital had not been very long known to people who had money to leave. The question as to whether the hospital was needed in its present position was answered by the large number of patients admitted. On a single day last August 101 new accidents had been brought for treatment. It was said that small hospitals were costly, but they could not get on without one at Poplar, and the expense was therefore unavoidable. Mr. Holland referred to the fallacy of comparing the cost per bed of one hospital with another without taking all the circumstances into account. Everything except soap was provided for the patients at Poplar, including tea, sugar, and butter. Some hospitals had no rent to pay. At this accidents hospital the patients were all very hungry men, with healthy appetites. He found that a patient, when his knee-cap was broken, eat as much or more than at other times, because he did not pay for his food in hospital. As long as the management of the funds rests with honourable men Mr. Holland thought that the governors might trust them to avoid extravagance, and not to fear comparison with other hospitals. (Applause.) In appointing Miss Bland to the matronship of the hospital a step had been taken which had proved an unmixed blessing, and nothing could be better than the nursing carried on at that hospital. With the unanimous consent of the working men on the committee, it was proposed to make a charge of 2d. to every out-patient, as the majority were quite able to meet so moderate a demand. With regard to the work of the nurses (a subject on which Mr. Holland said he was very keen), eleven hours' work a day and one half-day off duty every week ought to be practicable. If women were content to work longer hours, hospital managers ought not to be content to allow them to do so. A small number of extra nurses would be required to permit of the arrangement of time he advocated being universally adopted.

The resolution was seconded by Mr. J. G. Broodbank, who considered that the hospital was so efficiently and economically managed that no reduction of expenses was now possible.

The resolution was supported by Mr. Burdett, who remarked that the hospital would benefit by some directions as to the best way to reach it being included amongst the information given in the report. He had gone very carefully through the figures, and considered the cost per bed very creditable indeed; in fact, he thought it showed marvellous skill in management, considering that the institution was well-found in every department. They had the newest appliances, and he thought the hospital compared favourably with others in the matter of expenditure. Mr. Burdett said that the magnificent response to the chairman's special appeal had put fresh heart into people all over the country, and had re-acted on many institutions by showing that those who had money were willing to give liberally when the object for which funds were needed was a good one. The spirit which had shown itself at Poplar of late years was a great feature of the meetings, which seemed to be well attended, an active and lively interest being taken in the work of the committee. As one of the governors of the hospital, Mr. Burdett had great pleasure in supporting the resolution, congratulating Mr. Holland on his ingenuity in finding a succession of useful objects for which to appeal.

A resolution was carried for the dedication of a bed to the memory of Mr. F. Ernest Hills, the brother of the president.

Mr. Green, as a working man, said he felt the honour of being on the Managing Committee of such a hospital, and thanked the governors for electing him. He was warned that he would have to speak at their meeting, and he had spent the day in parading the neighbourhood collecting the experiences of those who had been patients in the wards. From all he heard the same story of plenty to eat and drink and good treatment. "The whole of those I called on throughout the whole piece said I might tell the same tale."

Mr. MacNulty, who spoke of himself as another working man from Canning Town, said what he and his class gave they were very jealous who had the handling of the money, and when satisfied that it was in good hands they were content to have it spent. He hoped their offerings would be still larger next year.

Dr. Corner proposed, and the Rev. A. E. Dalton seconded, a vote of thanks to Mr. John Aird, M.P., for presiding at the annual festival; to Mr. James Nourse for handsome donations; and the proprietors of the *Daily Chronicle* for their great help towards the building of the isolation block; and also to Mr. Henry C. Burdett and The Hospital newspaper for successful exertions to increase the Hospital Sunday Fund. Dr. Corner considered it a great triumph for them to have secured the approval of so fearless a critic as Mr. Burdett.

At the close of the meeting, Sir Herbert C. Perrott and

Dr. Freshfield joined the Hon. Sydney Holland and Lieut.-Colonel Feneran (hon. secretary) for the purpose of conferring (with the gracious permission of the Prince of Wales) the order of a Serving Sister of the Hospital of St. John of Jerusalem in England on Miss Bland, the matron. Miss Bland was one who held her duty in front of her—and did it, and the excellent reputation she had earned during many years at the great London Hospital as Sister of Gloucester Ward had been fully maintained since her appointment to the matronship at Poplar. The badge (a Maltese cross suspended from a black watered ribbon) was then pinned on by Dr. Freshfield, and Miss Bland acknowledged the honour which had been done to her in a few unaffected and well chosen words, concluding with a promise never to do anything to disgrace the Order to which she had been admitted.

### ROYAL LONDON OPHTHALMIC HOSPITAL. ANNUAL GENERAL MEETING.

At noon on March 24th, the annual general meeting of the governors of the Royal London Ophthalmic Hospital was held, and the chair was taken by Mr. C. Gordon, who moved the adoption of the report. He considered the general healthiness and the economy of the hospital very satisfactory, but lamented the great falling off in legacies. A large effort was required on the part of all concerned with regard to the large new site, and the building to be erected on it.

Mr. J. Tweedy referred to the inadequate recognition the great work of the hospital received in their rich City.

A vote of thanks to the editors of the metropolitan journals was proposed by the Rev. J. W. Pratt. They hoped for more favours from the Press, as it was desirable to give publicity to their plans for making the new hospital an absolutely first-class one.

The Chairman announced that £250 had been received from the Goldsmiths' Company since the subscriptions had been made up, and wished that other City companies would do as much for them.

The meeting concluded with thanks to Mr. Gordon, who had been associated with the hospital for over fifty years, and to the secretary for his well-known kindness and services extending over some twenty-five years.

The existing debt of £4,000 was mentioned with regret.

